# A retrospective study analyzing if lymph node ratio carbon nanoparticles predict stage III rectal cancer recurrence

**DOI:** 10.3389/fonc.2023.1238300

**Published:** 2023-10-30

**Authors:** Feng Pi, Gang Tang, Chaozheng Xie, Yukun Cao, Shilai Yang, Zhengqiang Wei

**Affiliations:** Department Of Gastrointestinal Surgery, The First Affiliated Hospital of Chongqing Medical University, Chongqing, China

**Keywords:** lymph node ratio, carbon nanoparticles, rectal cancer, recurrence, TNM

## Abstract

**Background:**

Lymph node ratio has garnered increasing attention as a prognostic marker for rectal cancer; however, few studies have investigated the relationship between lymph node ratio and rectal cancer recurrence. Additionally, Carbon Nanoparticle tracking is a safe and effective strategy for locating tumors and tracking lymph nodes. However, no studies have reported the relationship between Carbon Nanoparticles and rectal cancer recurrence.

**Methods:**

Patients with stage III rectal cancer who underwent radical resection between January 2016 and 2020 were analyzed. The primary outcome was tumor recurrence. 269 patients with stage III rectal cancer were included in this study. The effects of lymph node ratio, Carbon Nanoparticles, and other clinicopathological factors on rectal cancer recurrence were assessed using univariate, multivariate analyses and the t-test.

**Results:**

Univariate analysis determined tumor recurrence using cytokeratin 19 fragment, CA-199, CEA, N-stage, positive lymph nodes, total lymph nodes, and lymph node ratio(positive/total); with the lymph node ratio being the most relevant. Receiver operating characteristic (ROC) analysis determined lymph node ratio =0.38 as the optimal cutoff value. The analysis of lymph node ratio ≥0.38 and <0.38 showed statistical differences in three indicators: tumor recurrence, CEA, and use of Carbon Nanoparticles.

**Conclusion:**

Lymph node ratio is a strong predictor of stage III rectal cancer recurrence and may be considered for inclusion in future tumor-node-metastasis staging and stage III rectal cancer stratification. In addition, we found that Carbon Nanoparticles use significantly increased total lymph nodes and decreased lymph node ratio.

## 1 Introduction

Colorectal cancer remains a major clinical challenge, as most patients face recurrence even after undergoing curative surgery (1). It is firmly established that the prognosis of colorectal cancer is significantly correlated with the extent of tumor infiltration through the bowel wall and the presence of lymph node involvement. These two factors serve as the foundation for the development of the staging system for this disease (2). The current most widely accepted classification for colorectal cancer, as outlined by the American Joint Committee on Cancer (AJCC) and the International Union Against Cancer (UICC) tumor-node-metastasis (TNM) Classification of Malignant Tumors, classifies all colorectal cancers with lymph node metastasis as stage III (3), and it is recommended that at least 12 lymph nodes in each specimen be staged appropriately (4, 5).

Compared to colon cancer, the recurrence of rectal cancer seems to receive more surgical attention (6). The use of preoperative neoadjuvant therapy and total mesorectal excision has substantially reduced the local recurrence rate after rectal cancer surgery (7–12). However, rectal cancer poses a significant challenge in terms of diagnosis and prognosis when the lesion recurs in the narrow pelvic cavity owing to factors such as trophoblastic vessels and lymphatic drainage, especially when it is located in critical locations or involves important structures (13, 14). There are many staging systems for local recurrent rectal cancer, including the Leeds, MSKCC, and Mayo Clinic. Among these staging systems, MSKCC is most commonly used and can be divided into four types. The central type has a higher radical resection rate and yields better treatment outcomes for local recurrence following surgery. The anterior type is the second most effective; while for the posterior and lateral types, it is challenging to achieve R0 resection by reoperation resulting in less favorable treatment outcomes compared to the other types (15). Therefore, assessing the risk of rectal cancer recurrence has significant clinical implications, and early treatment interventions may significantly improve patient survival (16, 17).

The lymph node ratio (LNR), defined as the ratio of pathologically positive lymph nodes to total lymph nodes, has in recent years emerged as a strong predictor of cancer prognosis. The LNR has a significant impact on the survival curve of patients, underscoring its importance in predicting patient outcomes. This idea has been confirmed in studies on gastric, esophageal, breast, pancreatic, and colorectal cancers (18–22). The potential of the LNR as a prognostic marker in stage III CRC has been confirmed in numerous studies (1, 23, 24). Other clinicopathological or molecular markers have recently been considered promising predictors of prognosis in colorectal cancer, such as serum CEA, serum CA-199, serum cytokeratin 19 fragment, and degree of tumor differentiation (25, 26). However, because patients are at different disease stages, limited studies that have effectively integrated relevant factors affecting prognosis with tumor recurrence.

Carbon Nanoparticles (CNPs) have been widely used in our hospitals as novel tumor-staging markers (27, 28). Clinical studies have demonstrated that CNPs can facilitate accurate staging during lymph node dissection, thereby reducing operative time and intraoperative bleeding (29–31). Carbon Nanoparticle tracking is a safe and effective strategy for localizing tumors and tracking lymph nodes (32, 33). However, there are no studies on whether CNPs can reduce the risk of rectal cancer recurrence.

This study aimed to evaluate the effect of LNR and CNPs on recurrence in patients with stage III rectal cancer and to comprehensively discuss the findings based on the research.

## 2 Materials and methods

### 2.1 General data

The clinical data of 269 patients with rectal cancer who were admitted to The First Hospital of Chongqing Medical University for laparoscopic radical rectal cancer from January 2016 to January 2020 were retrospectively collected for inclusion in the study. These patients were in stage III according to the AJCC/UICC TNM malignancy classification, 8th edition.

#### 2.1.1 Inclusion criteria

1) The tumor was located in the rectum (defined as an intestinal tube 3 cm to 15 cm from the anus, and the distance was preoperatively determined by colonoscopy and MRI), and preoperative colonoscopy and pathological examination were clear for rectal cancer.
2) The tumor was confined to the intestinal wall and did not invade the posterior peritoneum or surrounding organs.

#### 2.1.2 Exclusion criteria

1) Extensive tumor infiltration; distant metastasis such as the liver and lungs.
2) Combined intestinal obstruction, history of abdominal surgery, extensive abdominal adhesions, and inability to undergo laparoscopic surgery for exploration.
3) Emergency surgery or laparoscopic intermediate open abdomen.
4) R1 or R2 resection (microscopic evidence of tumor infiltration at or within 1 mm from the resection margin, or unresected bulk residual tumor).
5) Synchronous colorectal tumors in different or the same segment.
6) Familial adenomatous polyposis, inflammatory bowel disease, or other primary malignancies.
7) Patients who were not regularly followed up at our hospital or missed their scheduled visits.

Under oncological principles, all patients in this study met the criteria for radical surgery, which is different because of the distance of the tumor from the anus (only MILES, Hartmann, and low anterior resection surgery were included in this study). The vast majority of patients with stage III rectal cancer who were included in the inclusion criteria received neoadjuvant therapy and received conventional adjuvant chemotherapy consultation; however, different chemotherapy regimens were possible. In addition, whether local or distant recurrence, we regard it as tumor recurrence.

### 2.2 Follow-up

We followed the patient for about 3 ½ years, The median follow-up was 31(25, 33) months. According to CSCO guidelines for colorectal cancer version 2022, all patients in this study were followed up every 3 months for 3 years, then every 6 months until 5 years after surgery, and once a year after 5 years. Each follow-up includes: abdominal physical examination (especially anal examination), blood tests, tumor markers (CEA, CA-199, Cytokeratin 19 fragment, etc.), and liver ultrasound. Chest + abdomen + pelvic CT (or CEA, if ultrasound is abnormal) done annually after surgery. One year after surgery, the colonoscopy is perfected. When recurrence is suspected, additional imaging tests are performed to clarify the diagnosis.

The primary endpoint of the study was tumor recurrence, which was defined as clear evidence of disease on imaging or biopsy. Cases that died during the follow-up period without imaging or biopsy evidence of disease were classified as “lost” cases and were excluded from this study.

Carbon Nanoparticles are injected in a way that is now widely accepted worldwide: 0.5 mL of Carbon Nanoparticles is injected through a rectoscope at three points around the primary tumor one day before surgery. (0.5 mL: 25 mg, Chongqing lummy Pharmaceutical Co.,Ltd) (28).

### 2.3 Data extraction

The following data were extracted from the hospital electronic database for analysis: age, gender, body mass index, distance of the tumor from the anus, cytokeratin 19 fragment, CA-199, CEA, Histological grade (well, moderate, and poor), TNM stage, positive lymph nodes, total lymph nodes, LNR, adjuvant therapy, postoperative chemotherapy, use of CNPs, and follow-up time.

The primary endpoint of the study was tumor recurrence, defined as clear evidence of the disease on imaging or biopsy. Patients who died during the follow-up period, without imaging or biopsy evidence of disease, were classified as “lost” cases and were excluded from this study.

### 2.4 Statistics

Data processing software was SPSS 26.0, and logistic regression analysis was used to assess the association of lymph node ratio, Using Carbon Nanoparticles and other pathology-related factors with stage III rectal cancer recurrence by univariate and multivariate analyses. This was followed by Receiver operator characteristic (ROC) analysis, the ROC analysis determines the optimal threshold by calculating the Youden’s index, which is calculated as sensitivity + specificity-1, and the value corresponding to the maximum of the Youden’s index is the determined cutoff value. According to the best cut-off value patients was divided into two groups, and the count data were expressed as % and carried out x2 test; the measurement data were expressed as mean ± standard deviation (x ± s), and t test was carried out to compare the two groups, and P<0.05 indicated that the difference was statistically significant. The two groups were also divided into two groups with or without the use of CNPs, and the t-test was conducted to compare the two groups, with P<0.05 indicating that the difference was statistically significant.

## 3 Results

During the five-year study period, a total of 269 patients diagnosed with stage III rectal cancer who underwent radical surgical resection with local lymph node dissection (meeting the inclusion and exclusion criteria) were included in this study. Detailed clinicopathological characteristics of these patients are shown in **Table 1**. The median age of the patients was 63 years (mean ± SD, 61.6 ± 12.4 years) and the median number of lymph nodes dissected was 13 (mean ± SD, 13.9 ± 6.1). The stage III CRC included 33 patients (12.3%) with stage IIIA, 145 (53.9%) with stage IIIB, and 91 (33.8%) with stage IIIC CRC.

Univariate analysis identified seven factors associated with recurrence of stage III rectal cancer: Cytokeratin 19 fragment, CA-199, CEA, N-Stage, positive lymph nodes, total lymph nodes, and LNR. By way of analysis, a higher total lymph nodes number was associated with a lower risk of recurrence, contrary to higher risk for positive LNs and LNR, and these data, as well as patient demographic and clinicopathological characteristics, are shown in **Table 1**.

**Table 1:** Demographic and clinicopathological features and univariate analysis associated with tumor recurrence.

	All patients (N = 269)*			Univariate analysis	
	N		OR	CI 95%	P
Age	62	31-90	0.988	0.962-1.015	0.393
Gender(Male/Female)	175/94	65/35	1.647	0.834-3.254	0.151
BMI	22.7	3.36	0.934	0.850-1.027	0.159
Distance of the tumor from the anus	8	1-15	0.994	0.900-1.096	0.899
Cytokeratin 19 fragment	3.5		1.098	1.027-1.175	0.006
CA-199	32.9		1.005	1.001-1.010	0.027
CEA	18.5		1.037	1.023-1.051	0.001
Histological grade			0.914	0.446-1.876	0.807
Well/Moderately differentiated	186	69			
Poorly differentiated	83	31			
T Stage			0.975	0.353-2.696	0.961
T1/T2	35	12			
T3/T4	238	88			
N Stage			2.431	1.223-4.831	0.011
N1	157	58			
N2	111	42			
Positive lymph nodes	3	1-6	1.135	1.058-1.219	0.001
Total lymph nodes	13	10-17	0.901	0.840-0.967	0.004
Lymph node ratio	0.35		37.052	10.298-133.320	0.001
Adjuvant therapy	229	85	0.988	0.385-2.532	0.980
Post-operative chemotherapy	259	96	0.688	0.141-3.363	0.644
Using Carbon Nanoparticles	93	35	0.680	0.323-1.432	0.310

*Data are number of patients (percentage), unless otherwise indicated. OR, hazard ratio. CI, confidence interval. Age, mean (range), years. BMI, body mass index; mean (standard deviation), kg/m2. Cytokeratin 19 fragment, median (interquartile range), ng/mL,CA-199=Carbohydrate antigen199, median (interquartile range),U/mL,CEA = carcinoembryonic antigen, median (interquartile range), ng/mL. All lymph nodes, median (interquartile range).

*Reference range: Cytokeratin 19 fragment(0-3.3ng/ml), CA-199(0-27.0U/ml, CEA(<5.2ng/ml).

Subsequently, we constructed two models: The first included all recurrence-related factors (p < 0.05) in a multivariate analysis, the results concluded that except for CEA (OR=1.028), none of the remaining six factors were independent prognostic factors (**Table 2**), but the LNR still showed the largest correlation (OR = 5.076). Considering the correlation between positive lymph nodes, total lymph nodes, and LNR, the second model analyzed only tumor markers and LNR, and the results showed that CEA and LNR emerged as independent factors associated with the recurrence of stage III rectal cancer (CEA p=0.001, hazard ratio 1.027, 95% confidence interval 1.012-1.042, LNR p=0.001, hazard ratio 18.473, 95% confidence interval 4.605-74.102); however, LNR still showed the largest correlation (OR=18.473) (**Table 3**).

**Table 2:** Multivariate analysis related to tumor recurrence*.

Covariate	OR	CI 95%	P
Cytokeratin 19 fragment	1.050	0.939-1.175	0.393
CA-199	1.003	0.997-1.010	0.321
CEA	1.028	1.012-1.044	0.001
N Stage	0.613	0.218-1.724	0.354
Positive lymph nodes	1.130	0.877-1.458	0.345
Total lymph nodes	0.865	0.718-1.042	0.126
Lymph node ratio	5.076	0.155-166.096	0.361

*Only covariates with trend-significance (p < 0.05) in univariate analysis were entered in multivariate analysis.

*OR, odds ratio; CI, confidence interval. Cytokeratin 19 fragment, ng/mL,CA-199=Carbohydrate antigen199, U/mL. CEA = carcinoembryonic antigen, ng/mL.

**Table 3:** Multivariate analysis related to tumor recurrence (factors relevant to LNR are excluded).

Covariate*	OR	CI 95%	P
Cytokeratin 19 fragment	1.044	0.931-1.171	0.464
CA-199	1.002	0.996-1.009	0.489
CEA	1.027	1.012-1.042	0.001
Lymph node ratio	18.473	4.605-74.102	0.001

*OR, odds ratio. CI, confidence interval. Cytokeratin 19 fragment, ng/mL, CA-199=Carbohydrate antigen199, U/mL, CEA = carcinoembryonic antigen, ng/mL.

**Figure 1:**
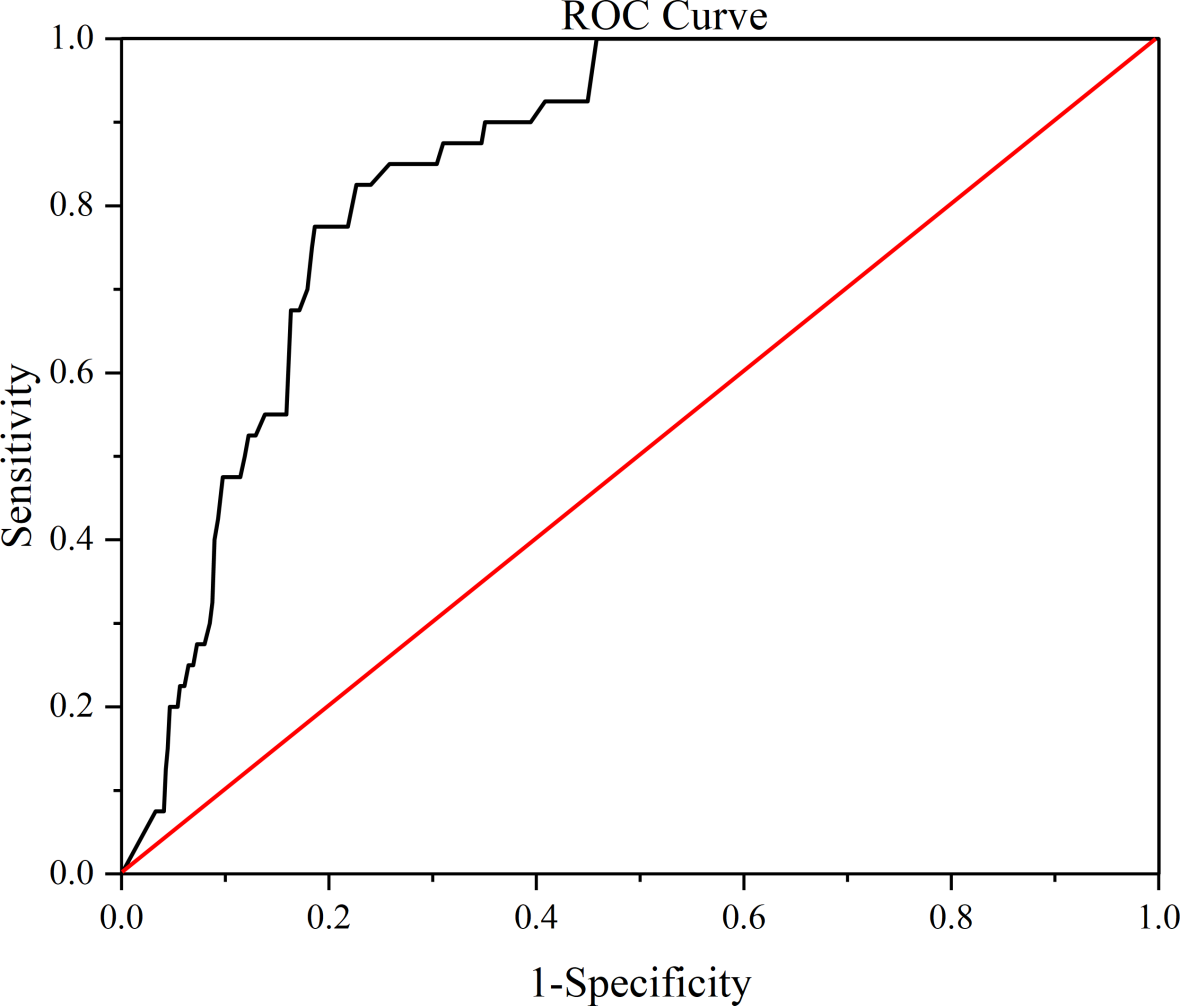
Receiver operator characteristic (ROC) analysis resulting curve.

The ROC analysis yields that the optimal cutoff value for predicting tumor recurrence exists for the lymph node ratio = 0.38 (**Figure 1**) (the optimal cutoff value has a sensitivity of 0.850, a specificity of 0.677), and patients were grouped according to the LNR on the basis of ROC analysis. There were 161 patients with a LNR <0.38 and 108 patients with a LNR ≥0.38. Comparing the two groups, the analysis revealed that there was no statistically significant difference between the baseline data of the two groups (**Table 4**). However, analysis of the factors related to recurrence showed that the proportion of recurrence was greater in the LNR≥0.38 group (31.2%) than in the LNR<0.38 group (3.7%), and the difference was statistically significant (P=0.001). But there was no significant difference in tumor recurrence time (P=0.885). Additionally, in the LNR≥0.38 group, the CEA levels (38.3 ± 107.88) were greater (10.12 ± 14.34) than the LNR <0.38 group, and the difference was statistically significant (P=0.048). Furthermore, the LNR≥0.38 group had a smaller proportion of patients using CNPs (25.9%) than the LNR<0.38 group (40.4%), and the difference was statistically significant (P=0.015) (**Table 4**). Subgroups with vs without CNPs use showed no statistically significant difference in baseline data between the two groups of patients (**Table 5**), and although the analysis yielded no statistically significant difference in tumor recurrence with CNPs, significantly increased the number of lymph nodes detected and decreased LNR. the use of CNPs significantly increased total lymph nodes and decreased LNR(**Table 5**). And LNR was confirmed to be closely related to the recurrence of stage III rectal cancer in our previous study, so we believe that the use of CNPs may be able to indirectly reduce the tumor recurrence rate by lowering the LNR, which deserves further in-depth study.

**Table 4:** Comparison of data between the two groups of patients (n=269).

Characteristics	1^a^(N=161)	2^b^(N=108)	Total(N=269)	pvalue
Age	61.82 ± 12.52	61.33 ± 12.38	61.62 ± 12.44	0.754
Gender				0.987
Male	104(64.6%)	71(65.7%)	175(65.0%)	
Female	57(35.4%)	37(34.3%)	94(35.0%)	
BMI	22.47 ± 3.44	22.93 ± 3.24	22.66 ± 3.36	0.273
Distance of the tumor from the anus	8.26 ± 3.38	7.58 ± 3.46	7.99 ± 3.42	0.107
Cytokeratin 19 fragment	3.10 ± 3.22	4.12 ± 6.36	3.51 ± 4.75	0.125
CA-199	29.34 ± 61.47	38.30 ± 118.56	32.94 ± 89.10	0.420
Histological grade				0.126
Well/Moderately differentiated	117(72.7%)	69(63.9%)	186(69.1%)	
Poorly differentiated	44(27.2%)	39(36.1%)	83(30.9%)	
T Stage				0.776
T1/T2	19(11.8%)	14(13.0%)	33(12.3%)	
T3/T4	142(88.2%)	94(87.0%)	236(87.7%)	
Adjuvant therapy	137(85.1%)	92(85.2%)	229(85.1%)	0.983
Post-operative chemotherapy	154(95.7%)	105(97.2%)	259(96.3%)	0.505
Tumor recurrence	6(3.7%)	34(31.2%)	40(14.9%)	0.001
Time to tumor recurrence*	12(8.5, 15.75)	12(9.75,14.25)	12(8.75,15.25)	0.885
CEA	10.12 ± 14.34	38.3 ± 107.88	18.54 ± 70.13	0.048
Using Carbon Nanoparticles	65(40.4%)	28(25.9%)	93(34.6%)	0.015

^a^Lymph node ratio<0.38.

^b^Lymph node ratio≥0.38.

*Age, M ± SD, years. BMI = body mass index, M ± SD, kg/m2. Cytokeratin 19 fragment, M ± SD, ng/mL, CA-199=Carbohydrate antigen199, M ± SD, U/mL, CEA = carcinoembryonic antigen, M ± SD, ng/mL, Positive lymph nodes, Total lymph nodes, Lymph node ratio, M ± SD. Reference range: Cytokeratin 19 fragment(0-3.3ng/ml), CA-199(0-27.0U/ml, CEA(<5.2ng/ml).

*Data are median time to tumor recurrence after surgery (25% percentile, 75% percentile).

**Table 5:** Comparison of data between the two groups of patients (n=269).

Characteristics*	1^a^(N=93)	2^b^(N=176)	Total(N=269)	pvalue
Age	62.09 ± 12.73	61.38 ± 12.32	61.62 ± 12.44	0.659
Gender				0.894
Male	61(65.6%)	114(64.8%)	175(65.0%)	
Female	32(34.4%)	62(35.2%)	94(35.0%)	
BMI	22.68 ± 3.68	22.65 ± 3.19	22.66 ± 3.36	0.944
Cytokeratin 19 fragment	3.44 ± 4.60	3.56 ± 4.84	3.51 ± 4.75	0.858
CA-199	36.27 ± 80.71	31.18 ± 93.41	32.94 ± 89.10	0.657
CEA	14.92 ± 22.34	20.45 ± 85.20	18.54 ± 70.13	0.539
Histological grade				0.524
Well/Moderately differentiated	62(66.7%)	124(70.5%)	186(69.1%)	
Poorly differentiated	31(33.3%)	52(29.5%)	83(30.9%)	
T Stage				0.188
T1/T2	15(16.1%)	18(10.2%)	33(12.3%)	
T3/T4	78(83.9%)	158(89.8%)	236(87.7%)	
Adjuvant therapy	79(84.9%)	150(85.2%)	229(85.1%)	0.951
Post-operative chemotherapy	88(94.6%)	171(97.2%)	259(96.3%)	0.343
Tumor recurrence	11(11.8%)	29(16.5%)	40(14.9%)	0.290
Time to tumor recurrence*	12(11.5,19.5)	12(8, 15)	12(8.75,15.25)	0.226
Positive lymph nodes	4.09 ± 3.86	4.53 ± 4.20	4.38 ± 4.08	0.393
Total lymph nodes	15.60 ± 6.10	12.97 ± 5.93	13.88 ± 6.11	0.001
Total lymph nodes≥12	70(75.2%)	91(51.7%)	161(60.0%)	0.001
Lymph node ratio	0.29 ± 0.25	0.52 ± 0.50	0.35 ± 0.27	0.007

^a^Using Carbon Nanoparticles.

^b^Without using Carbon Nanoparticles.

*Age, M ± SD, years. BMI = body mass index, M ± SD, kg/m2. Cytokeratin 19 fragment, M ± SD, ng/mL, CA-199=Carbohydrate antigen199, M ± SD, U/mL, CEA = carcinoembryonic antigen, M ± SD, ng/mL, Positive lymph nodes, Total lymph nodes, Lymph node ratio, M ± SD. Reference range: Cytokeratin 19 fragment(0-3.3ng/ml), CA-199(0-27.0U/ml, CEA(<5.2ng/ml).

*Data are median time to tumor recurrence after surgery (25% percentile, 75% percentile).

## 4 Discussion

Despite appropriate surgery and adjuvant therapy, the outcomes of patients with stage III rectal cancer remain unsatisfactory (34). The goals of the TNM classification of malignancies are to help clinicians plan treatment, provide prognostic indications for disease, assist in assessing treatment outcomes, and to facilitate the exchange of information between treatment centers (35). While the 7th edition of the AJCC and UICC colorectal cancer guidelines aimed to enhance the complexity of the staging system by creating N1 and N2 subcategories A and B, as well as by redefining subcategories for stage III cancer, these modifications proved to be insufficient due to the stage- independent differences in outcomes (36, 37). However, as recurrence is an important indicator for assessing patient prognosis, we need to improve the prognostic stratification based on the TNM staging system by including more indicators to assess the risk of recurrence in patients with stage III rectal cancer.

This retrospective study confirmed that LNR is one of the strongest predictors of recurrence in stage III rectal cancer. In addition to LNR, CEA also emerged as a prognostic factor for predicting the recurrence of stage III rectal cancer, although the correlation was less significant. For cancer, the prognostic significance of LNR has been described in four studies and it is considered a better prognostic factor than the number of metastatic lymph nodes (38–41), Of note, there are only few studies on LNR and the risk of recurrence and the comprehensive evaluation of these studies is inadequate. To better incorporate LNR into the TNM grading system, it must be reduced to a cutoff value rather than a continuous variable. We believe that the best way to determine the cutoff value is through ROC analysis.

Several studies have used this approach to assess the effect of LNR on survival curves in colon cancer. A study conducted by Galizia et al. investigated the disease-specific survival and found an optimal cutoff point of LNR=0.18 (42). Moreover, Greenberg et al. studied overall survival and found an optimal cutoff point of LNR=0.13 (24). In addition, Tiago et al. studied disease-free survival and overall survival and found an optimal cutoff point of LNR of 0.15 (23). It can be seen that the optimal cut-off points for the effect of LNR on the survival curves of colorectal cancer are all very stable, which gives us confidence in the sensitivity and accuracy of LNR for incorporating into the staging of colorectal cancer and assessing prognosis. While this current study evaluated the risk of recurrence in stage III rectal cancer and found the optimal cutoff point as LNR=0.38. It is important to note key factors that influenced our study findings: first, this study was exclusively conducted on rectal cancer and not colon cancer; and second, the study endpoint was tumor recurrence rather than the long-term prognosis of the patient. Moreover, some studies have shown differences in lymph node spread patterns between colon and rectal cancers, with a lower lymph node recovery rate in rectal surgical specimens and a higher number of metastatic lymph nodes, which also resulted in a higher lymph node metastasis rate (43). The results of the study proved that tumors of the colon and rectum cannot be generalized, and that patients can only benefit from a more stable and accurate LNR value if one is calculated. In conclusion, LNR was confirmed to be a strong predictor of recurrence in stage III rectal cancer, and we expect that this indicator will be added to the TNM classification in future versions to improve the TNM system.

With the rapid development of nanotechnology in medical technology, CNPs, one of the most representative nanomaterials, have been widely used for lymph node (LN) tracing in various surgeries (44). CNPs are widely used in our hospitals because they are selectively absorbed by lymphatic vessels and stain LNs black after injection into the submucosa surrounding the tumor. They also do not penetrate the capillaries and cannot enter the blood circulation because of the permeability differences between the lymphatic and blood systems (29). The use of CNPs has demonstrated promising results in enhancing the retrieval of more LNs (small and metastatic lymph nodes) (32), Therefore, we included CNPs as a factor in the univariate analysis to assess the risk of stage III rectal cancer recurrence. However, our results indicated no significant correlation between CNPs and the recurrence of stage III rectal cancer; but according to **Table 4**, the proportion of LNR ≥ 0.38 group using CNPs (25.9%) was smaller than that of LNR < 0.38 group (40.4%), and the difference was statistically significant (P=0.015). To further investigate the role of CNPs in predicting recurrence of stage III rectal cancer, we added a subgroup study of CNPs, which showed that CNPs significantly increased Total lymph nodes and decreased LNR, although the statistical difference with tumor recurrence was not significant. This was because CNPs had a positive effect on LN detection, making the denominator of the LNR larger, while the numerator was slightly larger or approximately unchanged. The Chinese Society of Clinical Oncology (CSCO) colorectal cancer guidelines clearly define that tumor clinical risk scores are closely related to positive lymph nodes in the primary tumor (45), Therefore, we believe that the use of CNPs to detect more lymph nodes can also indirectly detect and clear more positive lymph nodes, thus improving the quality of treatment while avoiding incorrect tumor staging, and perhaps even reducing stage III rectal cancer. The number of positive lymph nodes can also be indirectly detected and cleared, thus improving the quality of treatment while avoiding incorrect tumor staging and perhaps even reducing recurrence in patients with stage III rectal cancer. However, a possible selection bias due to the retrospective nature of the analysis is the main limitation of our study. Another limitation is low number of patients with tumor recurrence(n=40) and using CNPs(n=93). This hindered part of the statistical analysis. Therefore, further prospective studies are needed to validate the clinical potential of CNPs predicting recurrence in stage III rectal cancer.

There are still additional limitations to be addressed in this study. (1) The scope of this study was confined to stage III rectal cancer, precluding the opportunity to compare recurrence predictors between stage III colon and stage III rectal cancer; exploring such comparisons may yield valuable insights for improving TNM stratification, clinical diagnosis, and treatment. Thus, future studies addressing this is warranted. (2) The sample size of this study was only moderate and the study duration was limited; thus, to further advance the understanding of precise LNRs and the potential of CNPs, future studies should expand the scope through long-term investigations using larger sample sizes and multiple centers. (3) At present, this study did not take into account factors related to gene mutations, and there remains a need to include this index in future large-sample studies.

## 5 Conclusion

In conclusion, higher LNR was highly associated with stage III rectal cancer and was a strong predictor of the risk of stage III rectal cancer recurrence. The effect of CNPs on the LNR was also significant after grouping by LNR. CNPs use significantly increased Total lymph nodes and decreased LNR. Doing so CNPs can indirectly contribute to lower recurrence rates; however, further studies are warranted to investigate their role in the treatment of colorectal cancer.

## Data availability statement

The original contributions presented in the study are included in the article/supplementary material. Further inquiries can be directed to the corresponding author.

## Author contributions

Conceptualization: FP, GT. Data collection and analyses: CX, YC. Writing—original draft preparation: FP, SY. Writing—review and editing: ZW, had primary responsibility for final content. All authors contributed to the article and approved the submitted version.
